# 

**Published:** 1883-06

**Authors:** 


					﻿Vol. Ill,
JUNE, 1883.
NO. 6.
THE
HOMEOPATHIC pHY^lClAJI,
A MONTHLY JOURNAL OF MEDICAL SCIENCE.
“ Jtfog'na est veriias, et prcevalebit.”
k. J*. TjZEiel im:. id.,
EDITOR.
CONTENTS:
(See inside of Cover.)
PHILADELPHIA:
No. 3109 CHESTNUT STREET.
□ST S' "W ST O S. IC :
A. L. CHATTERTON, PUBLISHING COMPANY, Publisher’s Agents.
LONDON:
ALFRED HEATH & CO., Sole Agents for Great Britain,
114 Ebury Street, Eaton Square.
Yearly Subscription, $2.50, invariably in advance.
Entered at the Philadelphia Post-Office as Second-Class Mail Matter.
				

